# Atypical electrocardiographic findings in severe hyperkalemia with slow clinical course

**DOI:** 10.1002/jgf2.381

**Published:** 2020-10-03

**Authors:** Osamu Sasaki, Yozo Uriuda, Masaharu Shinkai, Hideki Sasaki

**Affiliations:** ^1^ Division of Internal Medicine Tokyo‐Shinagawa Hospital Tokyo Japan; ^2^ Department of Cardiovascular Surgery Tokyo Women's Medical University Hospital Tokyo Japan

**Keywords:** electrocardiography, hyperkalemia, slow clinical course

## Abstract

A 77‐year‐old woman walked into the emergency department with an episode of syncope and vomiting. She had visited at an orthopedic clinic with weakness of the lower extremities 6 weeks before, but cervical and lumbar MRI findings were unremarkable. Thereafter, she developed fingertip numbness and appetite loss at 7 and 3 days, respectively, before admission. She had been prescribed with RAS inhibitors for years. Electrocardiography while in the emergency department revealed bradycardia with normal QRS and a tented T wave. Laboratory findings revealed serum potassium 9.2 mEq/L. We discontinued RAS inhibitors and β‐blockers and added glucose‐insulin therapy. Thereafter, her general condition gradually recovered, and her symptoms completely disappeared. Elderly patients with chronic kidney disease treated with RAS inhibitors might develop slowly progressive symptoms of hyperkalemia. Electrocardiographic findings could be atypical and inconsistent with serum potassium values.

## Graphical abstract

Our manuscript is alert of “Atypical Electrocardiographic Findings in Severe Hyperkalemia with Slow Clinical Course.”

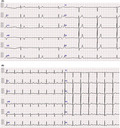

## 1 INTRODUCTION

Hyperkalemia is a life‐threatening condition that requires immediate treatment in an emergency situation. Factors associated with hyperkalemia include advanced age, decreased renal function, diabetes, and renin‐angiotensin system (RAS) inhibitors.^1^, ^2^ However, a diagnosis can be complicated when patients present with atypical clinical symptoms. We describe a patient who presented with slowly progressive symptoms and atypical electrocardiographic (ECG) findings.

## 2 CASE

**Figure 1:**
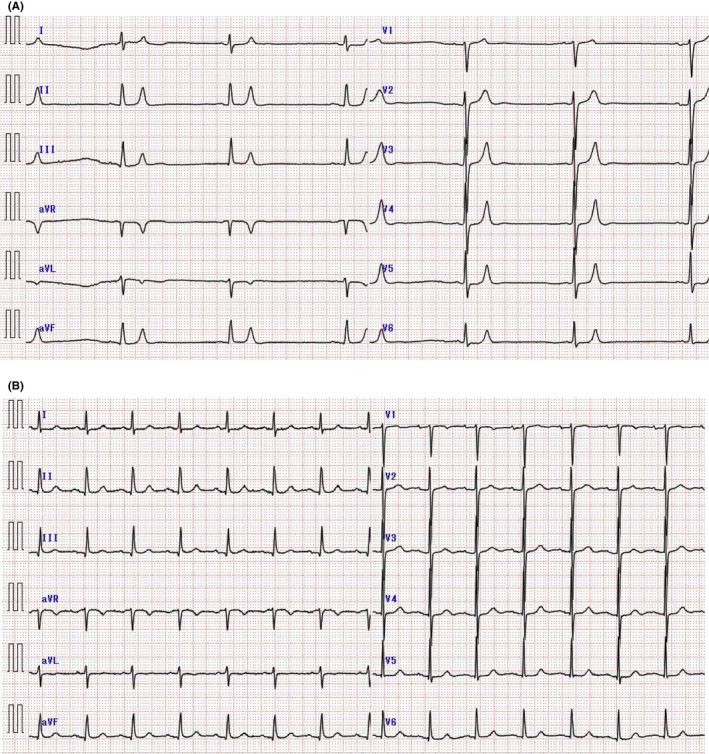
Electrocardiography and laboratory findings. A, Electrocardiography upon admission shows bradycardia (heart rate 37 bpm), decreased P‐wave amplitude, and tented T wave in limb and chest leads. Laboratory findings show serum potassium 9.2 mEq/L. B, Electrocardiography findings on hospital day 4 show normal sinus rhythm and improved T wave

A 77‐year‐old woman walked into the emergency department with an episode of syncope and vomiting. She had a 6‐week history of lower extremity weakness and had presented at an orthopedic clinic, where cervical and lumbar MRI findings were unremarkable. Five weeks later, she developed fingertip numbness followed by appetite loss 3 days before admission. The patient had been diagnosed with hypertension, hyperuricemia, and dyslipidemia. Aspirin 81 mg/d, telmisartan 40 mg/d, spironolactone 25 mg/d, allopurinol 100 mg/d, metoprolol tartrate 60 mg/d, and pravastatin sodium 10 mg/d were prescribed. She was conscious, with a temperature of 35.7°C; heart rate, 37 beats/min, and blood pressure, 185/54 mm Hg. Electrocardiography (ECG) in the emergency room revealed bradycardia, with a heart rate of 37 beats/min, decreased P‐wave amplitude, normal QRS width and a tall, tented T wave in limb and chest leads (Figure 1A). Laboratory findings revealed serum sodium 134 mEq/L, potassium 9.2 mEq/L, chloride 113 mEq/L, blood urea nitrogen 45 mg/dL, creatinine 2.2 mg/dL, and eGFR 17.7 mL/min/1.73 m^2^. Blood gas analysis (oxygen via nasal cannula 2 L/min) showed pH 7.234, pO2 118.6 mm Hg, pCO_2_ 32.1 mm Hg, HCO3‐ 13.1 mmol/L, BE −13.2, Hb 8.7 g/dL, TSH 0.76 μIU/mL, and Ca 9.5 mg/dL. Five months before admission, her serum creatinine was 1.5 mg/dL and eGFR was 26.2 mL/min/1.73 m^2^.

We considered that chronic kidney disease (CKD) and medication with telmisartan, spironolactone, and metoprolol tartrate had caused the hyperkalemia. We discontinued these medications. Furthermore, sodium bicarbonate, calcium gluconate and glucose‐insulin therapy was added. Her general condition gradually improved. By hospital day 4, her serum potassium improved to 4.5 mEq/L, ECG findings showed a normal sinus rhythm and an improved tented T wave (Figure 1B). Her symptoms of lower extremity weakness, fingertip numbness and appetite loss disappeared. Abdominal CT and upper/lower gastrointestinal endoscopy revealed no significant findings. Antihypertensive drugs were replaced with a calcium antagonist. She was discharged on hospital day 19.

## 3 DISCUSSION

This experience raised two important clinical issues.

Elderly patients with renal dysfunction who are under treatment with RAS inhibitors for hypertension might develop slowly progressive symptoms associated with hyperkalemia over several weeks. However, hyperkalemia has been typically diagnosed from a few to several days from the onset of symptoms.^3^, ^4^ Risk factors associated with hyperkalemia comprise renal dysfunction, diabetes, and medication with RAS inhibitors.^1^ Because RAS inhibitors suppress angiotensin‐II and aldosterone secretion, consequently inhibited potassium excretion leads to hyperkalemia. Our patient had renal dysfunction and had been prescribed with angiotensin‐II receptor blockers, RAS inhibitors, and β‐blockers for years. Her initial symptoms comprised lower extremity weakness that developed 6 weeks before admission. Fingertip numbness and appetite loss followed 5 weeks later. To conclude the correct diagnosis required 6 weeks because her symptoms were not specific to a single organ and the clinical course was slow. After glucose‐insulin therapy and discontinuing these medications, her serum potassium values gradually normalized, her symptoms such as lower extremity weakness, fingertip numbness and appetite loss completely disappeared; therefore, we concluded that hyperkalemia was the cause of the chronic symptoms that had persisted for 6 weeks. Elderly patients with renal dysfunction who are under treatment with RAS inhibitors and β‐blockers are susceptible to hyperkalemia and might present with an atypical clinical course that progresses over several weeks.

The ECG findings of patients with CKD with slow clinical symptoms of hyperkalemia might be atypical and inconsistent with serum potassium values. The ECG features of mild to moderate hyperkalemia include a tented T wave, prolonged PR, QRS, and QT intervals, and those of more severe hyperkalemia include sinoatrial and atrioventricular conduction disturbances that result in a widened QRS complex and sine‐wave morphology.^3^, ^4^, ^5^ The ECG findings of patients with serum potassium >8.0 mEq/L comprise an absent P‐wave, intraventricular block, bundle branch block, progressive widening of the QRS complex, a sine‐wave pattern, ventricular fibrillation, and asystole.^3^ However, ECG findings do not always indicate the severity of hyperkalemia.^3^, ^4^ Some studies^6^, ^7^ have found that the rate at which serum potassium increases might affect ECG findings, and patients with CKD appear to tolerate hyperkalemia better than patients without CKD. We speculated that the serum potassium level in this patient increased slowly and that the ECG findings were attenuated and atypical. Therefore, the initial ECG findings of our patient showed an essentially normal QRS despite a serum potassium value of 9.2 mEq/L. Thus, predicting serum potassium levels might be difficult.

Patients with CKD have 56% greater risk of all‐cause mortality compared with the general population.^8^ Hyperkalemia in patients with CKD increases the risk of ventricular arrhythmia, cardiac arrest, dialysis, and hospitalization.^9^ Severe hyperkalemia is quite prevalent in patients with CKD who experience out‐of‐hospital cardiac arrest.^10^ Our patient did not fall into cardiac arrest despite having severe hyperkalemia. The reason for this remains obscure. However, we speculate that a gradual increase in serum potassium might have been associated with the slow clinical course. Further data need to be accumulated to clarify this issue.

In conclusion, clinicians should be aware that some elderly patients with renal dysfunction who are medicated with agents that induce hyperkalemia might develop slowly progressive symptoms over several weeks, and that electrocardiographic findings could be atypical and inconsistent with serum potassium values.

## CONFLICT OF INTEREST

The authors have stated explicitly that there are no conflicts of interest in connection with this article.

## INFORMED CONSENT

Written informed consent for publication was obtained from the patient.
